# Effects of Lipid Solid Mass Fraction and Non-Lipid Solids on Crystallization Behaviors of Model Fats under High Pressure

**DOI:** 10.3390/molecules24152853

**Published:** 2019-08-06

**Authors:** Musfirah Zulkurnain, V.M. Balasubramaniam, Farnaz Maleky

**Affiliations:** 1 Food Technology Division, School of Industrial Technology, Universiti Sains Malaysia, Minden 11800, Penang, Malaysia; 2 Department of Food Science and Technology, The Ohio State University, 2015 Fyffe Court, Columbus, OH 43210, USA; 3 Department of Food Agricultural and Biological Engineering, The Ohio State University, 2015 Fyffe Court, Columbus, OH 43210, USA

**Keywords:** lipid gel, fully hydrogenated soybean oil, microstructure, additive effects, high pressure, heat compression

## Abstract

Different fractions of fully hydrogenated soybean oil (FHSBO) in soybean oil (10–30% *w/w*) and the addition of 1% salt (sodium chloride) were used to investigate the effect of high-pressure treatments (HP) on the crystallization behaviors and physical properties of the binary mixtures. Sample microstructure, solid fat content (SFC), thermal and rheological properties were analyzed and compared against a control sample (crystallized under atmospheric condition). The crystallization temperature (*T_s_*) of all model fats under isobaric conditions increased quadratically with pressure until reaching a pressure threshold. As a result of this change, the sample induction time of crystallization (*t_c_*) shifted from a range of 2.74–0.82 min to 0.72–0.43 min when sample crystallized above the pressure threshold under adiabatic conditions. At the high solid mass fraction, the addition of salt reduced the pressure threshold to induce crystallization during adiabatic compression. An increase in pressure significantly reduced mean cluster diameter in relation to the reduction of *t_c_* regardless of the solid mass fraction. In contrast, the sample macrostructural properties (SFC, storage modulus) were influenced more significantly by solid mass fractions rather than pressure levels. The creation of lipid gel was observed in the HP samples at 10% FHSBO. The changes in crystallization behaviors indicated that high-pressure treatments were more likely to influence crystallization mechanisms at low solid mass fraction.

## 1. Introduction

In food products, fats and oils contribute to the structural and sensory characteristics of the product that are intrinsically linked to consumer acceptability. The crystallization kinetics of lipids greatly influences the final development of the crystalline structure, polymorphic forms, and their rheological properties [1]. Hence, the crystallization and modifications of fats aim to obtain lipid bases with acceptable characteristics specially when there are zero trans fats, lower saturated fats, and higher mono and polyunsaturated fatty acids content. Recently, studies indicated the use of hard fats, in particular fully hydrogenated oils (FHO) fats, as potential crystallization modifiers of liquid oils and other fats [2,3,4]. FHO consists of homogeneous saturated triacylglycerols (TAGs) of a high melting point (>40 °C), such as tristearin, and acts as a structuring element that accelerates crystallization in systems composed of low and medium melting TAGs [5]. When diluted with oil, the high melting point TAGs form a three-dimensional network of fat crystals inside liquid oils that have the ability to extend the plastic range of food products [6,7]. 

However, the naturally occurring crystallization on these lipids usually favor formation of the most thermodynamically stable polymorphic form β on dilution. This may result in high melting points and large spherulitic crystals with poor macroscopic properties [3,8]. Hence, it is recommended that the appropriate processing conditions, such as cooling rate, agitation (shear), pressurization and processing temperature, are applied to affect the crystallization behavior of TAG molecules [9,10,11,12]. High pressure treatments of lipids have been investigated to gain a fundamental understanding of phase transition [13,14,15] and its applications on fatty acids [16,17] and fats [18,19,20,21]. Interestingly, studies have also documented that minor components which are either present indigenously or added in fats (such as emulsifiers, mono- and diacylglycerols, and non-fat ingredients) can influence the system’s crystallization and final properties [9,10]. Despite molecular dissimilarity to fats, foreign crystals such as inorganic (magnesium silicate, carbon nanotube, graphite) and organic (terephthalic acid) materials have been shown to promote fat crystallization [22,23]. It has also been shown that the effect of processing conditions is more pronounced at low solid mass fraction where the capacity of the crystalline fraction of the TAGs to form a solid network in the liquid oil plays crucial roles in the structuring ability [22,24].

Recently our group investigated the crystallization behavior of different binary mixtures of fully hydrogenated soybean oil and soybean oil under high pressure treatments, consisting of adiabatic compression to a targeted pressure followed by isobaric cooling [25]. The findings revealed distinct crystallization properties at nano-, micro- and macroscale when the crystallization started during adiabatic compressions and continued during isobaric cooling. Regardless of pressure levels and maximum temperatures achieved under pressure, the rapid onset of crystallization during adiabatic compression resulted in smaller homogeneous crystal structures [25]. This finding agreed with other studies on pressurization of lipids that showed that forced convection during compression prior to the onset of phase transition can elucidate the enhancement of structural modification via an improvement in the kinetic mobility of TAGs during the initial stage of lipid crystallization (nucleation) [26].

To further investigate the effects of high pressure treatments on lipids, here we studied the crystallization behavior of the same lipid systems (a mixture of fully hydrogenated soybean oil in soybean oil) in the presence of additives (as a non-lipid solid particle). Paying attention to the effects of solid mass fraction and salt particles as a non-lipid solid particle, we monitored the crystallization behaviors during the compression step to identify and quantify structure characteristics responsible for differences in macroscopic properties formed under high-pressure treatments.

## 2. Results and Discussion

### 2.1. Crystallization Temperature (T_s_) and Pressure Threshold

**Figure 1:**
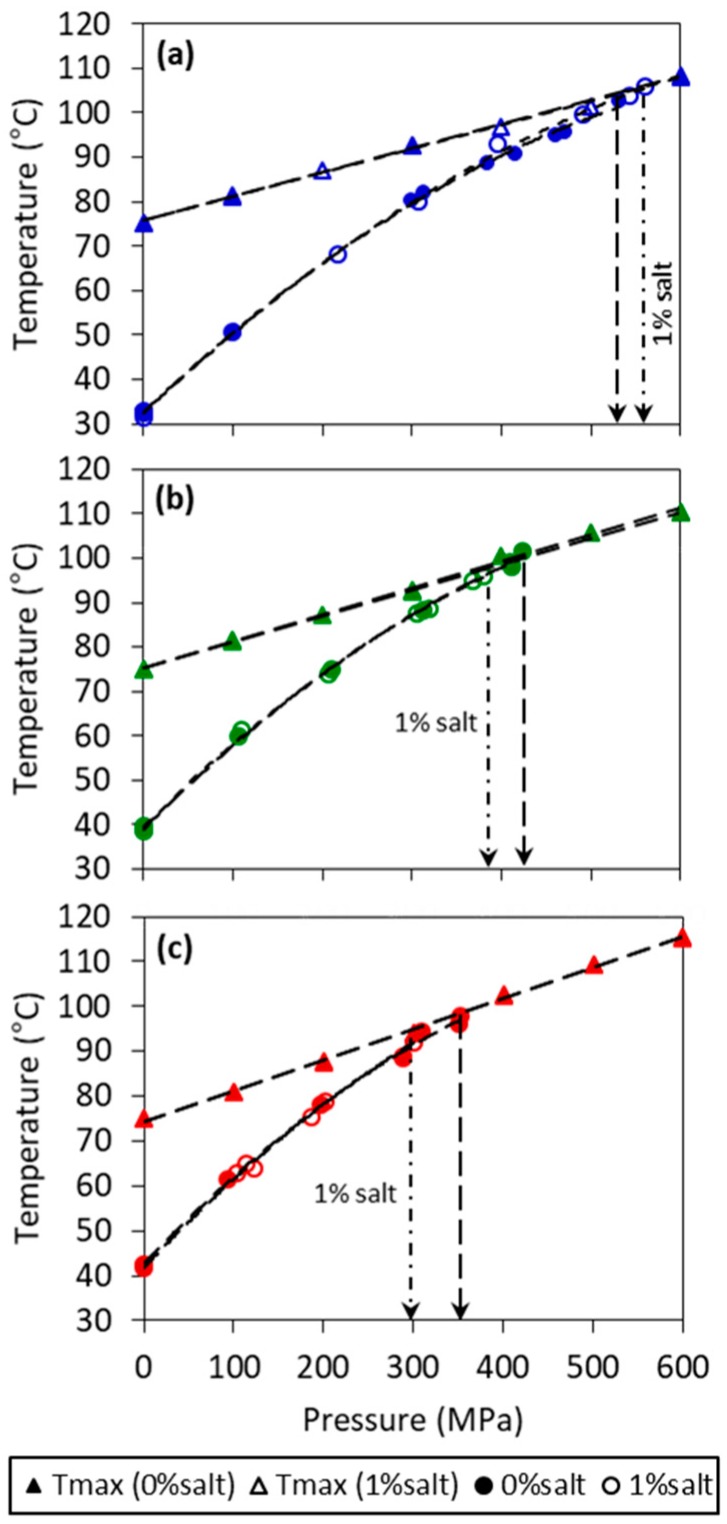
Maximum temperature under pressure (*T_max_*) (triangle symbol) and phase diagram (circle) of model fats at (**a**) 10%, (**b**) 20%, and (**c**) 30% of fully hydrogenated soybean oil (FHSBO) without (filled symbol) and with 1% salt (empty symbol). Vertical arrows show pressure threshold where crystallization temperature (*T_s_*) crosses *T_max_*.

The effects of pressure and salt on crystallization temperature (*T_s_*) of three model fats made of 10, 20, 30% fully hydrogenated soybean oil (FHSBO) in soybean oil are shown in Figure 1. The *T_s_* of the model fats increased in a quadratic manner with an increase in pressure and was well fitted into a quadratic function with a regression coefficient of R^2^ > 0.99 and the sum of squared errors of prediction (SSE) < 6.94, as shown in Table 1. A quadratic relation of the melting temperature with pressure has been reported by Ferstl et al (2010) as up to 450 MPa for triolein [18]. Several studies have shown that high pressure treatments promote crystallization of lipids by increasing the melting temperature between 10 to 24 °C/100 MPa [13,14,16,18]. The phase boundary coefficient, *dT_m_/dP* of homogeneous fatty acids have been reported at 12.7 °C/100 MPa and 23.9 °C/100 MPa for α and ß polymorphic forms, respectively [27]. In the absence of melting point under pressure, a phase transition diagram based on the crystallization temperature can be a close substitute to understand the fundamental response of lipid materials during high pressure processing [15,19]. As shown from the values of coefficient *a_1_* in Table 1, an increase in the solid mass fraction from 10% to 30% FHSBO increased the phase boundary coefficients based on the *T_s_* from 18.4 to 20.3 and 21.2 °C/100 MPa for the model fats at 10%, 20%, and 30% FHSBO, respectively. The table also shows that the addition of salt did not significantly affect these values.

At an initial temperature (*T_i_*) of 75 °C (a temperature higher than the fat models melting point at atmospheric condition), the maximum temperature under pressure (*T_max_*) achieved during compression step increased linearly with increase in pressure levels from 100 MPa to 600 MPa, (shown in Figure 1). The linear relation of *T_max_* with pressure is confirmed by showing a high regression coefficient for all mass fraction (*R^2^* > 0.99). The linear coefficient (*b_0_*) of the slope of *T_max_* shows small increments with the increase in solid mass fraction from 5.5 to 6.9 °C/100 MPa for 10% and 30% FHSBO, respectively (Table 1), which was not affected by the addition of salt. As indicated by vertical arrows in Figure 1, the phase boundaries of all model fats were truncated when the *T_max_* crossed *T_s_*, as the onset of crystallization shifted from the isobaric condition (during the pressure holding step) to adiabatic condition (during the rapid compression step). Instantaneous phase transition was evident from a strong increase in temperature during adiabatic compression when the crystallization happened before reaching *T_max_*. This set of data are shown in Figure S1 (Supplementary Materials). The occurrence of the sudden increase in temperature during the compression of lipid materials has been reported in other studies and is linked to the emission of the latent heat of crystallization and the existence of small crystalline bodies [17,28]. 

Table 1 also reports the pressure threshold of 531.7, 423.6, and 351.0 MPa for model fats without salt at 10%, 20%, and 30% FHSBO, respectively. The increase in solid mass fraction reduced the pressure threshold required to induce crystallization because lower unsaturated fatty acid content makes less free volume available for compression [29]. Interestingly, the addition of 1% salt increased the pressure threshold for 10% FHSBO sample from 531.7 MPa to 560.1 MPa but reduced the pressure threshold from 423.6 to 379.6 MPa for 20% FHSBO and from 351.0 to 300.5 MPa for 30% FHSBO samples. When the supersaturation was low at low solid mass fraction (10% FHSBO), longer pressurization may impart forced convection in the liquid state before phase transition take place, which was observed by Tefelski et al. (2011) during the pressurization of oleic acid [26]. Whether the addition of salt leads to convection effects that affect nuclei formation is not clear.

### 2.2. Induction Time of Crystallization (t_c_)

Further, the effects of salt particle during crystallization was evaluated from the induction time of crystallization shown in Table 2. For atmospheric crystallization, the increase in the solid mass fraction from 10% to 30% FHSBO significantly reduced the induction time from 6.5 to 3.6 min, respectively. High pressure crystallization resulted in significantly lower induction time compared to the atmospheric crystallization. An increase in pressure significantly decreased the induction time, as reported previously by Ferstl et al. (2011) [29]. At 100 MPa, the induction time during the isobaric condition (between 2.33 to 2.74 min) was not affected by the increase in solid mass fraction or salt addition. At 300 MPa, the addition of salt significantly reduced the induction time of crystallization for the 30% FHSBO model fat from 0.92 min during isobaric condition to 0.46 min during adiabatic condition, as the pressure threshold had been reached. At 600 MPa, when crystallization happened under an adiabatic condition, the increase in solid mass fraction (from 10% to 30% FHSBO) significantly reduced the induction time of crystallization (from 0.66 to 0.46 min). This is directly related to the reduction of the pressure threshold with the increase in the solid mass fraction (Table 1). 

### 2.3. Melting Properties of the Crystallizaed Model Fats

**Figure 2:**
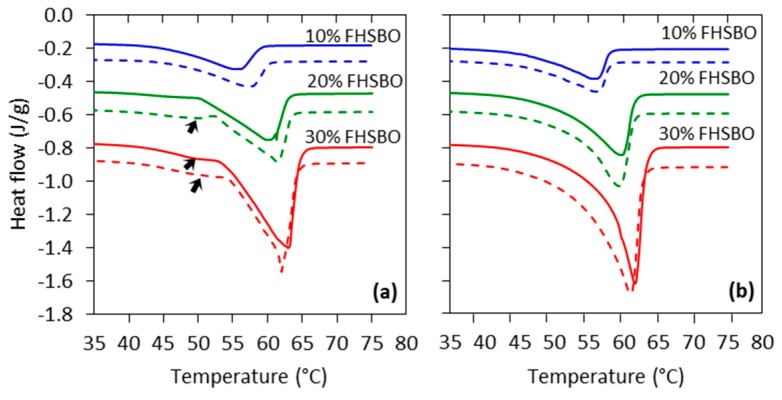
Melting thermograms of model fats at 10%, 20%, and 30% fully hydrogenated soybean oil (FHSBO) without (─) and with 1% salt (---) crystallized under (**a**) atmospheric pressure and (**b**) 600 MPa.

The fatty acid composition of all model fats, without and with salt, is reported in Table 3. The addition of salt did not affect the fatty acid composition of the model fats. To explore the effects of the pressure level on the samples, their melting profile was studied immediately after the processing, when the crystalline solid-state structure remained unaffected. Figure 2 shows a representative of the melting thermograms of samples crystallized under atmospheric conditions compared to the high pressure treatment at 600 MPa. The melting point (*T_m_*), onset of melting (*T_o_*), and specific enthalpy of melting (Δ*H_m_*) of samples crystallized at 0.1, 100, 300, and 600 MPa are shown in Table 4. The melting curves of the atmospheric crystallized samples at 30% FHSBO and 20% FHSBO (with 1% salt) show a shoulder (indicated by arrows in Figure 2a) in addition to a broad prominent peak (β phase), which suggests the presence of a β’ polymorph phase in these samples [8,25]. The result of the 30% FHSBO is in agreement with the Ribeiro et al. who reported the existence of a β’ phase in the mixture of FHSBO and soybean oil for samples containing more that 30% FHSBO [8]. In accordance with Ostwald’s step rule, studies have shown that the crystallization of most fully hydrogenated oils favors the formation of a metastable α phase which is kinetically favored at the beginning of phase transition [30,31]. It is also shown that blending FHO with high amount of oil (>80%) allows the adoption of a more thermodynamically stable polymorphic form because a reduction in the viscosity of the mixtures enhances TAG diffusion [8,30,32]. This kinetic of crystallization may vary when other non-fat particles are added to the system, when the model fat at 20% FHSBO (with 1% salt) still shows formation of the β’ phase. The presence of foreign particles may increase the rate of crystallization as it kinetically favors the formation of the less stable polymorphic phase when TAG molecules are incorporated into the crystalline surface very quickly, and therefore imperfectly. 

Despite the atmospheric crystallized samples, all of the high pressure crystallized samples (without and with 1% salt) showed a single peak with a sharp and narrow melting exotherm that suggest the existence of only the most stable β polymorphic phase in the high pressure crystallized samples. This is in agreement with the previous study of 30% FHSBO without the addition of salt [25]. The reduction in free space under high pressure increases the degree of supersaturation and thus, increases the thermodynamic driving force for polymorphic transformation [33]. Moreover, the high pressure treatments significantly reduced *T_m_* and increased Δ*H_m_* of the samples compared to the atmospheric crystallization (Table 4). Increase in pressure levels from 100 to 600 MPa further reduced *T_m_* of the samples except for 20% FHSBO. The reduction of the pressured sample melting points can be explained by the depression of their freezing points (colligative effect) due to the higher solubility of TAGs in samples crystallized at high pressure levels [30]. These effects of TAGs solubility were not observed on the *T_m_* of pressured fat with 30% FHSBO due to differences in the sample’s initial temperature (*T_i_*) of the pressurization [23].

Further analysis of Figure 2 (dotted thermogram in Figure 2b) shows that salt addition resulted in the formation of a broader exothermic peak (with higher Δ*H_m_*) for samples containing 20% and 30% FHSBO. The comparison of the samples Δ*H_m_* is shown with star symbols in Table 4. The increase in Δ*H_m_* may infer the formation of a higher amount of crystals in the samples that is in agreement with the significant increment of these sample’s solid fat content (SFC) shown in Table 5 [25]. This finding may translate into differences in crystallization behavior and the resulting network properties of the samples. 

### 2.4. Samples Solid Fat Content (SFC), Shear Storage Modulus, and Microstructure

The effects of atmospheric (0.1 MPa) and high pressure treatments (100, 300, and 600 MPa) on the solid fat content (SFC) of the model fats, without and with 1% salt, are shown in Table 5. Although there were no significant differences observed between the SFC values of the 10% FHSBO samples, sample homogeneity was different in this set of samples (data are not shown here). For 10% FHSBO, the atmospheric crystallized samples showed sedimentation of large fat crystals at the bottom of the glass tube upon storage for 24 hours at 20 °C, while all high pressure crystallized samples maintained their gel texture. This observation indicates a clear phase separation of the atmospheric sample’s solid crystals and liquid oil, in which the samples can be classified as having low plasticity and low resistance to oiling-out [34,35,36]. This could be confirmed by the low *G’* values (15.1 Pa) measured for this set of samples, as shown in Table 6. Although the addition of salt increased the sample *G’* to 21.4 Pa, sample storage modulus remained lower than their loss modulus (*G”* = 44.1 ± 3.7 Pa). This indicates that the dispersed low concentration of solid crystallites was not able to bind the liquid oil and oil remains as the continuous phase to induce liquid-like properties to the system [35]. In contrast, Table 6 shows significantly higher *G’* values (from 2.9 × 10^4^ to 9.4 × 10^4^ Pa) for the pressurized samples at 10% FHSBO. An increase in pressure levels significantly increased their solid-like behavior with a significant increase in the G’ values. They formed a translucent lipid gel with a homogeneous distribution of high density small stable β crystals that helps them hold their shape and solid texture.

**Figure 3:**
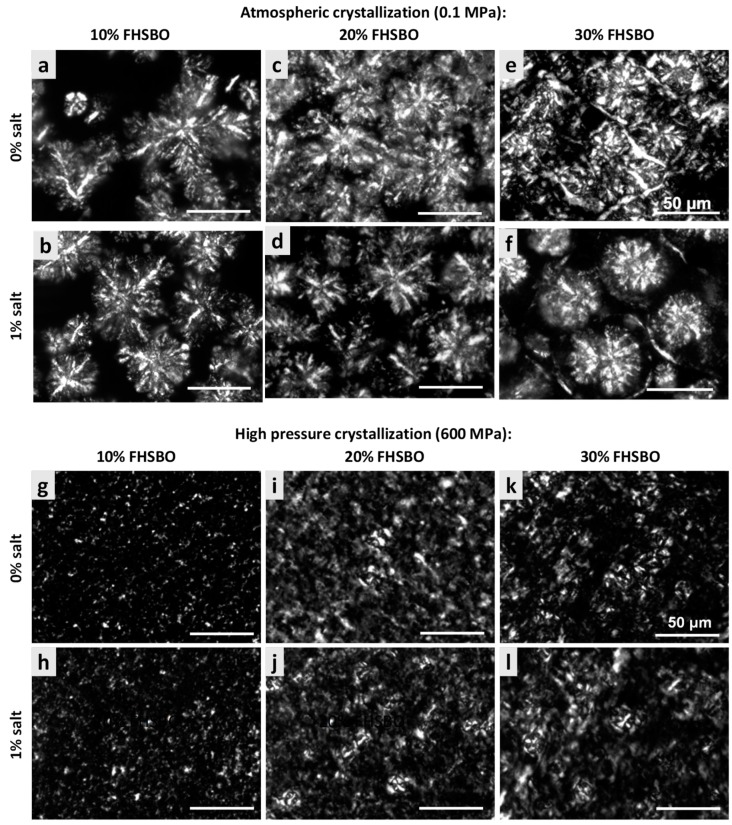
Polarized light microscopy (PLM) micrographs (500× magnification) of model fats of 10%, 20%, and 30% FHSBO, without salt and with 1% salt, crystallized at atmospheric condition (**a**–**f**) and under high pressure treatment at 600 MPa (**g**–**l**).

**Figure 4:**
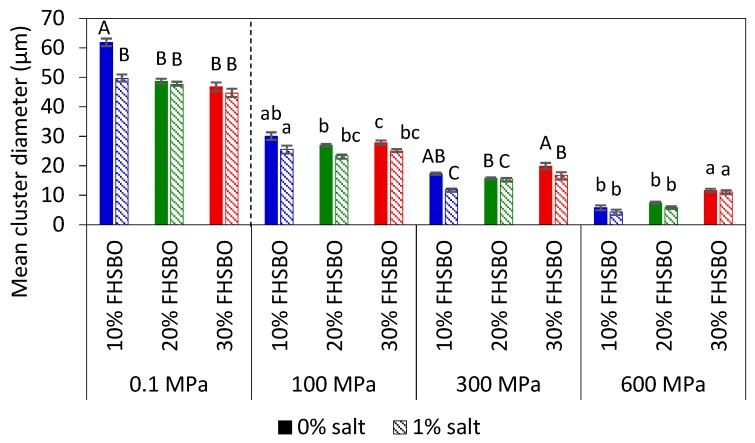
Mean cluster diameter of 10%, 20%, and 30% fully hydrogenated soybean oil (FHSBO), without and with 1% salt, crystallized at different pressure levels. Different upper-case or lower-case show significant difference (*p* < 0.05) between samples at different percentage of FHSBO.

Figure 3 represents and compares the microstructures of the samples, without and with 1% salt, crystallized under atmospheric and high pressure treatment at 600 MPa. The mean cluster diameter of all the crystallized samples is summarized in Figure 4. At 10% FHSBO, the microstructure of the atmospheric crystallized samples is spherulitic in nature with well-defined dendritic aggregations of large crystallites. They have largest mean cluster diameter with dimension of 61.8 ± 1.3 μm. The growth of the spherulites was not radial, resulting in characteristic Maltese-cross shapes, parallel with observations reported by Omonov et al. (2010) [35]. Bouzidi et al. (2013) and Omonov et al. (2010) reported that a high portion of liquid oil allows high melting TAGs (tristearin) to continuously grow into larger spherulites that branch out into dendrites until the concentration of high melting TAGs diminishes [35,37]. Under a polarized light microscope, the crystallites were seen segregated from the oil with the largest open dendrites and looser network, as reported by Omonov et al. (2010) [35]. This allows the liquid phase to leave the solid network more easily compared to the denser fibrillar network at higher solid mass fractions. Under an atmospheric condition, the addition of salt to 10% FHSBO resulted in the formation of denser and smaller branched dendrites that reduced the size of the spherulites to a smaller dimension at 49.6 ± 11.5 μm (Figure 4).

In contrast, PLM of 10% FHSBO samples crystallized under high pressure at 600 MPa (Figure 3g–h) shows unique microstructures of non-spherulitic behavior. The primary microstructural elements did not show characteristics of crystalline fibers like dendritic or needle. Rather, the microstructural elements, which originate from different individual nucleus, are small and uniformly distributed and responsible for their gel-like behaviors (formed during the rapid compression step at 600 MPa). A similar creation of β-fat gel made of binary mixtures of high-melting and low-melting fats has been reported by Higaki et al. (2004) via melt-mediated transformation [24]. Occasionally, a small crystal cluster may form with the average diameter of 5.8 ± 1.0 μm (Figure 4). As shown in Figure 4, the mean cluster diameter increased with an increase in pressure levels by about 5 times to 30.1 ± 1.3 μm at 100 MPa. This might be explained by the slower nucleation and its subsequent growth under isobaric conditions (at lower pressure levels) at the expense of the smaller surrounding crystals. Interestingly, the addition of salt shows a significant reduction in the mean cluster diameter under this condition at 100 and 300 MPa. 

At 20% FHSBO, the high pressure crystallization increased the SFC from 19.3% to the range of 20.4–20.6% for non-salted samples. The addition of 1% salt further increased the SFC values of the high pressure crystallized samples from 19.6% to the range of 21.2–21.3%. This is supported by the differences in the fat crystal structure formed under atmospheric (Figure 3c–d) versus high pressure treatments (Figure 3i–j). Although both samples have the physical state of a semi-solid fat, the atmospheric crystallization resulted in highly contrasted PLM micrographs that represent a high molecular order in the crystal structure [35]. The mean cluster diameter of these 20% FHSBO samples are between 44.6 to 48.6 μm (Figure 4) and are not affected by the addition of 1% salt. The increase in solid mass fraction from 10% to 20% FHSBO resulted in more densely packed crystal clusters, thus limiting radial growth of the spherulites by the adjacent agglomerates when formed slowly under atmospheric crystallization [38]. 

On the other hand, the PLM of high pressure crystallized samples at 20% FHSBO (Figure 3i–j) showed circular clusters of poorly defined boundaries embedded in high number of small primary microstructural elements, due to higher nucleation rates, and a faster initial overall crystal growth. The mean cluster diameters of these samples were significantly smaller compared to the atmospheric crystalized samples ranging from 26.9 ± 0.4 μm at 100 MPa to 5.8 ± 0.5 μm at 600 MPa (Figure 4). Similar to samples at 10% FHSBO, an increase in pressure significantly reduced the mean cluster diameter of the samples with the greatest impact seen at 600 MPa and the addition of salt reduced the diameters at low pressure levels. Interestingly, the 20% FHSBO high pressure crystallized samples showed comparable *G’* with the atmospheric crystallized samples ranged between 4.2 × 10^5^ (0.1 MPa) to 5.9 × 10^5^ Pa (600 MPa). The addition of salt significantly increased the G’ of the high pressure crystallized sample at 600 MPa to 6.4 × 10^5^ Pa when the crystallization took place during an adiabatic condition. This was also supported by the increment of the samples Δ*H_m_* showing higher crystal formation. 

At 30% FHSBO, pressurization with the addition of salt significantly increased the SFC of the samples at all pressure levels to the range between 31.3–32.4% (denoted with star symbol in Table 5). The sample’s *G’* are comparable between the atmospheric crystallization (2.4 × 10^6^ Pa) and the high pressure crystallization ranged between 2.1 × 10^6^ Pa (100 MPa) to 2.4 × 10^6^ Pa (600 MPa), as shown in Table 6. However, the atmospheric crystallized samples were brittle in nature, as given by a smaller linear viscoelastic region (LVR) reported earlier [12]. The LVR is defined as the region where a fat material is able to hold its texture before the bond between fat crystals network is broken. All of the high pressure crystallized samples have longer LVR than the atmospheric crystallized samples, suggesting a stronger bond between their fat crystal networks. Solid networks based on a small crystal network have increased specific surface areas that increase contact with the oil and tend to be firmer [39]. The addition of salt significantly increased the storage modulus of the sample at 600 MPa at 2.7 × 10^6^ Pa. Like SFC, the effect of salt on the *G’* of the high pressure crystallized samples was significant when the crystallization happened during adiabatic compression at 600 MPa. 

Although similar in textural properties at macroscale, the microstructures of the atmospheric crystallized samples were significantly larger compared to the high pressured crystallized samples, as shown in Figure 4. The mean cluster diameter of the atmospheric crystals was between 44.7 ± 1.3 to 46.8 ± 1.5 μm and were at least twice larger than the high-pressure crystallized samples between 27.9 ± 0.7 μm at 100 MPa to 11.1 ± 0.7 μm at 600 MPa. As shown in Figure 4, the atmospheric crystallized samples show a crystal network of discrete spherulites of large structures that contributes to their brittle texture. However, the PLM micrograph of samples crystallized with the addition of 1% salt shows a lower contrast which is well correlated with the reduction of Δ*H_m_* of these samples (Table 4). As discussed earlier, the presence of foreign particles may suggest the formation of less perfect crystals when TAG molecules are incorporated into the crystalline surface quickly. 

For the high-pressure crystallized samples, increase in pressure significantly reduced the mean cluster diameter regardless of the addition of salt. This shows that the effect of pressure levels is more prominent than solid mass fraction at the microstructure level, in contrast to the macrostructural properties. Interestingly, the influence of solid mass fraction was significant at 600 MPa when the mean cluster diameters were reduced with the reduction in FHSBO fraction from 30% to 10% in contrary to the increment of their induction time of crystallization (Table 2). High pressure crystallization at 600 MPa resulted in unique microstructures that constituted of large numbers of very small size and uniform distribution of microstructural elements between 0.1 to 5 μm, as reported in earlier work of binary mixtures at 30% FHSBO [25]. In static crystallization, an increase in SFC is expected to increase the viscosity of the melt that may limit mass transfer and crystal growth [40]. However, the opposite results may suggest a different nucleation mechanism and crystal growth during the dynamic compression step.

## 3. Materials and Methods 

### 3.1. Sample Preparation 

Model fats of different solid mass fractions were prepared by blending 10%, 20%, and 30% *w/w* fully hydrogenated soybean oil (FHSBO) with soybean oil (SBO). Iodized salt (Morton Salt, Chicago, IL, USA) was powdered and passed through a 630-mesh sieve. The lipid mixtures were heated at 90 °C for 15 min under constant stirring to erase their crystal memory. An amount of 1% *w/w* salt was added to the melted mixture at 90 °C under constant vigorous stirring. Both the mixtures, with and without salt, were dispensed carefully while under heating and constant stirring, to ensure the samples homogeneity, in a5-mL polypropylene syringe (Becton Dickinson and Co., Franklin Lakes, NJ, USA) with a movable plunger and rubber stopper. This sample carrier was insulated with two layers of tape (CVS Pharmacy Inc., Woonsocket, RI, USA). 

### 3.2. Fatty Acid Composition

The fatty acid composition of the model fats was determined according to AOCS Ce 1-62 standard method [41]. 

### 3.3. High-Pressure Crystallization Experiments 

The high pressure experiments were conducted using a high-pressure kinetics tester (PT-1, Avure Technologies, Kent, WA, USA) with a 54-mL high-pressure chamber (0.02 m internal diameter). A high-pressure intensifier (M-340A, Flow International, Kent, WA, USA) was used to achieve a pressure level up to 700 MPa at the rate of 12 MPa/s. The decompression time was at 4 s regardless of holding pressure. Propylene glycol (Brenntag North America Inc., Reading, PA, USA) was used as the pressure-transmitting fluid. The pressure and temperature were monitored and recorded every 1 second using DasyLab software (Version 7.00.04, National Instruments Corp., Austin, TX, USA). 

Prior to high-pressure treatment, samples were thermally equilibrated in a water bath at 90 °C to erase crystal memory. A K-type thermocouple (KMTSS-062G-12, Omega Engineering, Stamford, CT, USA) was positioned in the middle of the sample carrier. The direct contact of the thermocouple junction with the sample enabled temperature measurement in the sample during pressure treatment. Samples were pressurized over a range of pressures between 100 MPa and 600 MPa from a fixed initial temperature (*T_i_*) of 75 °C, and the maximum temperature under pressure (*T_max_*) was recorded. The *T_i_* was set higher than the melting temperatures of the model fats to ensure the samples were in melt state and to avoid any chance of nucleation in the sample prior to pressurization. Then, the samples were held under the targeted pressure and allowed to cool for 10 min by maintaining the pressure’s chamber at 30 °C for model fats of 20% and 30% FHSBO and 25 °C for model fat of 10% FHSBO. Finally, the samples were depressurized to the atmospheric pressure in 4 seconds and taken out for analysis. All high-pressure crystallization experiments were conducted in duplicate, and the crystallized samples were analyzed within one hour after the treatment. The solid crystallized samples were carefully removed from the sample carrier and sliced into three parts. Physical and structural analysis of the samples were made using the sample taken from the center slice. The samples were stored at 20 °C for 24 hours for analysis that demand further observations.

### 3.4. Atmospheric Crystallization Experiments (Control Sample) 

Samples were equilibrated in a water bath at 75 °C and crystallized at atmospheric pressure (0.1 MPa). The experiments were conducted by immersing the samples in a temperature-controlled bath for 30 min at 30 °C for fat mixtures of 20% and 30% FHSBO and 25 °C for fat mixtures of 10% FHSBO, with and without salt. The setup conditions were aimed to keep the degree of supercooling (Δ*T_s_*) which is the temperature difference between crystallization temperature (at atmospheric condition, *T_s_*) and temperature of the pressure medium comparatively close between samples at mean value of 11.4 ± 1.7 °C. As shown in Table 1, the difference between the crystallization temperature of samples at 10% and 30% FHSBO was higher (~10 °C) compared to samples at 20% and 30% FHSBO (~3 °C). Sample temperature was recorded every 1 second using DasyLab software (Version 7.00.04, National Instruments Corp., Austin, TX, USA). The control experiments were conducted in duplicate and the crystallized samples were analyzed within one hour after the treatment. Except for the sample at 10% FHSBO (liquid state), the solid crystallized samples were carefully removed from the sample carrier and sliced into three parts. Sampling for physical and structural analysis of the samples were conducted using the center part of the slice.

### 3.5. Determination of Crystallization Temperature and Induction Time 

Using the principles of thermometry analysis [42], the first derivative of the sample’s temperature history (dT/dt) was plotted against pressure and temperature to identify the induction time of crystallization (*t_c_*), and crystallization temperature (*T_s_*), and pressure. The crystallization temperature (*T_s_*) is defined as the peak maxima of the first derivative of the sample’s temperature history for samples crystallized during adiabatic compression (>300 MPa), as shown in Figure S1 (Supporting Information). For samples crystallized at atmospheric pressure and during isobaric cooling, the *T_s_* is corresponded to the peak minima of the first derivative of the sample’s temperature history. The induction time was defined as the time taken to reach *T_s_* from the start of pressurization. Samples *T_s_* and the corresponding pressure *(P)* can be related using a simple polynomial expression as follow:

*T_s_* = a_0_*P*^2^ + a_1_*P* + a_2_ (1)

The coefficients of regression of Equation (1), a_0_, a_1_, and a_2_, was estimated by regression analysis using MINITAB version 16 statistical software (Minitab, Inc., State College, PA, USA). On the other hand, the relationship between the maximum temperature under pressure (*T_max_*) and pressure was expressed as follow:

*T_max_* = b_0_*P* + b_1_ (2)

where the regression coefficient of Equation (2), b_0_ and b_1_, was estimated by regression analysis using MINITAB version 16 statistical software (Minitab, Inc., State College, PA, USA).

### 3.6. Solid Fat Content (SFC)

About 3 g of the crystallized sample was placed into glass vials (Sigma-Aldrich, St. Louis, MO, USA) and the sample’s SFC was measured by pulse nuclear magnetic resonance (p-NMR) spectrometer (Bruker Minispec mq20, Bruker Corporation, Billerica, MA, USA) at room temperature. The reported data corresponds to the average of five individual measurements.

### 3.7. Thermal Analysis

The thermal behaviors of the model fats at different solid mass fraction of FHSBO (10%, 20%, 30% *w/w*), with and without salt, was analyzed by differential scanning calorimetry (DSC) equipped with a refrigerated cooling system (Q2000, TA Instrument, New Castle, DE, USA) [23]. About 10 mg of each model fat was hermetically sealed in an aluminum pan, and an empty pan was used as a reference. 

The thermal behavior of the high-pressure and atmospheric crystallized samples was studied by melting each sample (10 mg) in a sealed hermetic aluminum pan. The sample pan was heated from 20 to 80 °C at 5 °C/min. Sample melting temperatures (*T_m_*), the onset of melting (*T_o_*) and the enthalpy of melting (Δ*H_m_*) were determined using TA Universal Analysis 2000 (Advantage Software v5.5.24/Universal Analysis Software, TA Instrument, New Castle, DE, USA). 

### 3.8. Small Amplitude Oscillatory Rheological Measurement 

Dynamic oscillatory measurements were conducted using a strain control rheometer (MCR 302, Anton Paar, Graz, Austria). Plate-plate geometry (20 mm) with sandblasted fixtures were used with normal force set at 3 ± 0.5 N for all measurements. The loading protocol consisted of applying an increasing normal force control (1–10 N), allowing the sample to relax the axial force to a constant value over a zero strain relaxation test for 15 min. Amplitude sweeps were performed imposing a constant frequency (ω) of 6.28 rad/s. The storage modulus (*G′*) and loss modulus (*G″*) were determined within the linear viscoelastic region (LVR). 

### 3.9. Microstructural Analysis

A polarized light microscope (Axio Imager.M2m, Carl Zeiss Microscopy GmbH, Jena, Germany) with high-resolution CCD camera was used to analyze the microstructure of the crystallized samples. To obtain satisfactory reproducibility of the microscope slides, a definite amount (~1 mg) of the crystallized sample was placed on each microscope slide, spread in all directions and gently secured with cover slips. Images were acquired using 20× and 50× objective lens and AxioVision software (Carl Zeiss Microscopy GmbH, Jena, Germany). 

The threshold of the microstructure images at 20x magnification were manually set, converted to grayscale and analyzed for particle size distribution using Image J software (Version 1.50b, National Institutes of Health, Bethesda, MD, USA). The particle size distributions were fitted using the Gaussian function in PeakFit software (Seasolve, Framingham, MA, USA) to determine mean particle equivalent diameter and standard deviation from full width at half maximum of the Gaussian peak. The crystal cluster size was measured using the Circle feature of AxioVision software on images at 50× magnification. The program assumes a circular geometry and obtains the square root of the quotient of the area to π. Since the crystal lattice of sodium chloride is cubic and highly symmetric, it is not birefringence in nature. Thus, under polarized light microscopy, the salt particles appeared as black particles embedded in the crystalline matrix [38]. They can be clearly distinguished from the fat matrix by applying partial polarization. 

### 3.10. Statistical Analysis

Reported values correspond to means with standard deviations of the measurements. Statistical analysis between means was carried out by one-way ANOVA (*p* < 0.05) with Tukey’s multiple comparisons as a post-test (*p* < 0.05) using MINITAB version 16 statistical software (Minitab, Inc., State College, PA, USA). 

## 4. Conclusions

The crystallization of binary mixtures of FHSBO and soybean oil under pressure were evaluated at different solid mass fraction and the addition of 1% salt. The investigated model fats showed a significant nonlinear increase of the crystallization temperature with pressure up to the pressure threshold when the onset of crystallization shifted from isobaric to adiabatic condition as the crystallization temperature reached the maximum temperature under pressure. The crystallization behaviors of different mixtures observed showed the domination effects of pressure levels on the induction time and size of the microstructure. At high-pressure levels, when crystallization was induced during an adiabatic condition, the short induction time resulted in the formation of a high number of small and homogenously distributed fat crystal structures. However, macrostructural properties were strongly influenced by the solid mass fraction of the mixtures. The enhancement effects of the high-pressure treatments on the SFC and shear storage modulus were observed at low solid mass fraction (10% FHSBO) with the creation of lipid gel. The promotional effects of salt were detected on the SFC, thermal, and rheological properties at high solid mass fraction especially beyond the pressure threshold. The results indicate that high pressure treatment could be used as a novel physical process to modify the structural properties of FHSBO/soybean oil blends to produce healthier lipid sources such as trans-free and low-saturated fat, tailoring to their specific functional properties and applications.

## Figures and Tables

**Table 1:** Quadratic coefficient relating crystallization temperature (*T_s_*) with pressure and linear coefficient of maximum temperature (*T_max_*) with pressure for different model fats at 10%, 20%, and 30% fully hydrogenated soybean oil (FHSBO) without and with 1% salt. SSE is sum of squared of errors of prediction.

Coefficients	10% FHSBO		20% FHSBO		30% FHSBO	
	0% Salt	1% Salt	0% Salt	1% Salt	0% Salt	1% Salt
*a*_0_ (°C/(100 MPa)^2^)	−1.17	−1.06	−1.36	−1.34	−1.57	−1.65
*a*_1_ (°C/100 MPa)	18.4	19.0	20.3	19.9	21.2	21.1
*a*_2_ (°C)	32.72	32.45	38.94	39.57	41.86	42.70
*R* ^2^	0.999	0.999	0.999	0.999	0.999	0.998
SSE	6.94	6.49	3.63	4.54	5.3	8.63
*b*_0_ (°C/100 MPa)	5.5	5.4	6.0	5.8	6.9	6.9
*R*^2^ (*T_max_*)	0.999	0.996	0.997	0.999	0.998	0.998
Pressure threshold (MPa)	531.7	560.1	423.6	351.0	351.0	300.5

**Table 2:** Induction time of crystallization for fat models with 10%, 20%, and 30% fully hydrogenated soybean oil (FHSBO) without and with 1% salt.

Pressure (MPa)	Induction time of crystallization ^1^ (min)					
	10% FHSBO		20% FHSBO		30% FHSBO	
	0% Salt	1% Salt	0% Salt	1% Salt	0% Salt	1% Salt
**0.1**	6.18 ± 0.2 ^Ai^	6.47 ± 0.0 ^ai^	4.53 ± 0.3 ^Aii^	4.20 ± 0.2 ^aii^	3.69 ± 0.1 ^Aiii^	3.68 ± 0.3 ^aiii^
**100**	2.69 ± 0.3 ^Bi^	2.74 ± 0.1 ^bi^	2.72 ± 0.1 ^Bi^	2.61 ± 0.0 ^bi^	2.49 ± 0.3 ^Bi^	2.33 ± 0.1 ^bi^
**300**	1.39 ± 0.1 ^Ci^	1.44 ± 0.1 ^ci^	0.85 ± 0.0 ^Cii^	0.82 ± 0.0 ^cii^	0.92 ± 0.2 ^Cii^	0.49 ± 0.1 ^ciii*^
**600**	0.66 ± 0.1 ^Di^	0.72 ± 0.0 ^di^	0.49 ± 0.0 ^Dii^	0.44 ± 0.0 ^dii^	0.46 ± 0.1 ^Dii^	0.43 ± 0.0 ^cii^

^1^ Means in the same column followed by different upper-case letters are significantly different (*p* < 0.05) between pressure levels. Means in the same row followed by different Roman letters are significantly different (*p* < 0.05) between solid mass fractions. Star symbol (*) denotes significant difference between means in the same row for samples without and with addition of 1% salt.

**Table 3:** Fatty acid composition of fully hydrogenated soybean oil (FHSBO) and model fats at different percentages of FHSBO, without and with 1% salt.

Fatty Acid (%)	FHSBO	FHSBO:SBO (*w*/*w*%)					
		10:90		20:80		30:70	
		0% Salt	1% Salt	0% Salt	1% Salt	0% Salt	1% Salt
Linoleic acid (C18:2n6)	-	49.6	50.6	44.0	44.6	39.4	39.0
Stearic acid (C18:0)	91.1	11.8	11.4	20.4	20.2	29.0	28.8
Oleic acid (C18:1n9)	-	20.6	21.9	18.4	19.1	15.9	16.3
Palmitic acid (C16:0)	8.9	11.1	10.8	11.2	10.9	11.1	10.6
γ-Linolenic acid (C18:3n6)	-	4.8	4.9	4.2	4.8	4.5	4.9
Linolenic acid (C18:3n3)	-	0.2	0.2	0.2	0.2	0.2	0.3

**Table 4:** Melting point (*T_m_*), onset of melting (*T_o_*), and specific enthalpy of melting (Δ*H_m_*) of 10%, 20%, and 30% fully hydrogenated soybean oil (FHSBO), without and with 1% salt, crystallized at different pressure levels.

**Melting Point ^1^ (*T_m_*, °C)**						
**Pressure (MPa)**	**10% FHSBO**		**20% FHSBO**		**30% FHSBO**	
	**0% Salt**	**1% Salt**	**0% Salt**	**1% Salt**	**0% Salt**	**1% Salt**
0.1	57.10 ± 0.2 ^A^	57.64 ± 0.6 ^a^	60.61 ± 0.6 ^I^	61.23 ± 0.2 ^i^	62.74 ± 0.3 ^A^	62.11 ± 0.4 ^a^
100	56.31 ± 0.1 ^B^	56.89 ± 0.1 ^b^	59.29 ± 0.5 ^II^	59.34 ± 0.4 ^ii^	62.11 ± 0.3 ^B^	61.59 ± 0.3 ^ab^
300	56.37 ± 0.2 ^B^	56.30 ± 0.1 ^c^	59.48 ± 0.1 ^II^	59.58 ± 0.2 ^ii^	61.57 ± 0.1 ^C^	61.40 ± 0.1 ^b^
600	55.90 ± 0.2 ^C^	55.84 ± 0.1 ^d^	59.38 ± 0.2 ^II^	59.32 ± 0.3 ^ii^	61.83 ± 0.2 ^BC^	61.08 ± 0.2 ^b*^
**Onset of Melting ^1^ (*T_o_*, °C)**						
**Pressure (MPa)**	**10% FHSBO**		**20% FHSBO**		**30% FHSBO**	
	**0% Salt**	**1% Salt**	**0% Salt**	**1% Salt**	**0% Salt**	**1% Salt**
0.1	46.02 ± 0.5 ^A^	45.81 ± 0.4 ^a^	52.72 ± 2.0 ^A^	53.25 ± 2.3 ^a^	53.41 ± 0.8 ^A^	54.11 ± 0.9 ^ab^
100	46.37 ± 0.7 ^AB^	48.20 ± 0.1 ^d*^	52.50 ± 0.9 ^A^	52.14 ± 2.1 ^a^	56.12 ± 0.7 ^B^	53.98 ± 0.8 ^ab*^
300	47.23 ± 0.9 ^AB^	47.85 ± 0.2 ^c^	52.50 ± 1.6 ^A^	52.39 ± 2.0 ^a^	57.07 ± 1.6 ^B^	54.00 ± 0.2 ^a*^
600	47.72 ± 1.0 ^B^	46.87 ± 0.4 ^b^	51.76 ± 1.1 ^A^	53.58 ± 1.5 ^a*^	56.96 ± 1.0 ^B^	55.21 ± 0.6 ^b*^
**Specific Enthalpy of Melting ^1^ (Δ*H_m_*, J/g)**						
**Pressure (MPa)**	**10% FHSBO**		**20% FHSBO**		**30% FHSBO**	
	**0% Salt**	**1% Salt**	**0% Salt**	**1% Salt**	**0% Salt**	**1% Salt**
0.1	17.67 ± 0.1 ^A^	16.86 ± 0.6 ^a*^	34.64 ± 1.1 ^A^	35.16 ± 0.4 ^a^	58.45 ± 2.1 ^A^	52.58 ± 2.3 ^a*^
100	18.86 ± 0.6 ^B^	16.62 ± 0.6 ^a*^	37.93 ± 1.0 ^B^	39.92 ± 1.6 ^b*^	60.88 ± 2.7 ^AB^	61.66 ± 2.7 ^b^
300	18.87 ± 0.5 ^B^	18.79 ± 0.2 ^b^	38.64 ± 2.0 ^B^	40.49 ± 2.3 ^b^	60.66 ± 1.6 ^AB^	67.51 ± 1.2 ^b*^
600	18.80 ± 0.3 ^B^	19.11 ± 0.2 ^b^	37.89 ± 1.0 ^B^	40.39 ± 1.0 ^b*^	63.34 ± 0.3 ^B^	70.61 ± 3.2 ^b*^

^1^ Means in the same column followed by different upper-case letters are significantly different (*p* < 0.05). Star symbol (*) denotes significant differences between means in the same row for samples, without and with addition of 1% salt.

**Table 5:** Solid fat content (SFC) of model fats at 10%, 20%, and 30% fully hydrogenated soybean oil (FHSBO), without and with 1% salt, crystallized at different pressure levels.

Pressure (MPa)	Solid Fat Content ^1^ (SFC, %)					
	10% FHSBO		20% FHSBO		30% FHSBO	
	0% Salt	1% Salt	0% Salt	1% Salt	0% Salt	1% Salt
0.1	10.92 ± 0.12 ^A^	10.97 ± 0.11 ^a^	19.31 ± 0.24 ^II^	19.58 ± 0.31 ^ii^	28.78 ± 0.15 ^B^	20.07 ± 0.46 ^b^
100	11.07 ± 0.04 ^A^	11.15 ± 0.12 ^a^	20.54 ± 0.05 ^I^	21.21 ± 0.04 ^i*^	29.83 ± 0.37 ^A^	31.28 ± 0.48 ^a*^
300	11.12 ± 0.03 ^A^	11.18 ± 0.17 ^a^	20.58 ± 0.05 ^I^	21.23 ± 0.06 ^i*^	29.66 ± 0.08 ^A^	32.38 ± 0.20 ^a*^
600	11.17 ± 0.05 ^A^	11.19 ± 0.17 ^a^	20.44 ± 0.04 ^I^	20.33 ± 0.07 ^i*^	29.78 ± 0.07 ^A^	31.63 ± 0.27 ^a*^

^1^ Means in the same column followed by different upper-case, lower-case, and Roman letters are significantly different (*p* < 0.05) between pressure levels. Star symbol (*) denotes significant difference between means in the same row for samples without and with addition of 1% salt.

**Table 6:** Shear storage modulus (*G’*) of model fats at 10%, 20%, and 30% fully hydrogenated soybean oil (FHSBO), without and with 1% salt, crystallized at different pressure levels.

Pressure (MPa)	Shear Storage Modulus ^1^ (*G’*, Pa)					
	10% FHSBO		20% FHSBO		30% FHSBO	
	0% Salt	1% Salt	0% Salt	1% Salt	0% Salt	1% Salt
0.1	15.1 ± 3.2 ^C^	21.4 ± 2.0 ^d^	4.1 × 10^5^ ± 1.2 × 10^5 I^	4.5 × 10^5^ ± 9.9 × 10^4 ii^	2.4 × 10^6^ ± 2.0 × 10^5 A^	2.3 × 10^6^ ± 3.2 × 10^5 ab^
100	2.9 × 10^4^ ± 4.3 × 10^3 B^	3.3 × 10^4^ ± 1.4 × 10^3 c^	4.7 × 10^5^ ± 5.4 × 10^4 I^	3.7 × 10^5^ ± 3.7 × 10^4 ii*^	2.1 × 10^6^ ± 7.1 × 10^4 B^	2.2 × 10^6^ ± 4.0 × 10^4 b^
300	5.3 × 10^4^ ± 1.8 × 10^3 A^	5.7 × 10^4^ ± 2.7 × 10^3 b^	4.6 × 10^5^ ± 1.2 × 10^4 I^	5.0 × 10^5^ ± 2.9 × 10^4 i,ii^	2.3 × 10^6^ ± 9.4 × 10^4 AB^	2.5 × 10^6^ ± 1.6 × 10^5 ab^
600	6.1 × 10^4^ ± 2.4 × 10^3 A^	9.4 × 10^4^ ± 1.3 × 10^4 a^	5.9 × 10^5^ ± 2.9 × 10^4 I^	6.4 × 10^5^ ± 1.4 × 10^4 i*^	2.4 × 10^6^ ± 1.2 × 10^5 A^	2.7 × 10^6^ ± 1.1 × 10^5 a*^

^1^ Means in the same column followed by different upper-case, lower-case, and Roman letters are significantly different (*p* < 0.05) between pressure levels. Star symbol (*) denotes significant difference between means in the same row for samples without and with addition of 1% salt.

## Supplementary Materials

The following are available online at https://www.mdpi.com/1420-3049/24/15/2853/s1, Figure S1: First derivative of temperature as a function of pressure during adiabatic compression at 600 MPa. Arrow indicates crystallization temperature (*T_c_*) at local maximum of the first derivative of temperature curve.
