# Supplementary figures and images for: Effects of Lipid Solid Mass Fraction and Non-Lipid Solids on Crystallization Behaviors of Model Fats under High Pressure
Source: Molecules. 2019 Aug 6;24(15):2853. doi: 10.3390/molecules24152853 (PMC6696334; doi:10.3390/molecules24152853)

Supplementary Materials  
Figure S1

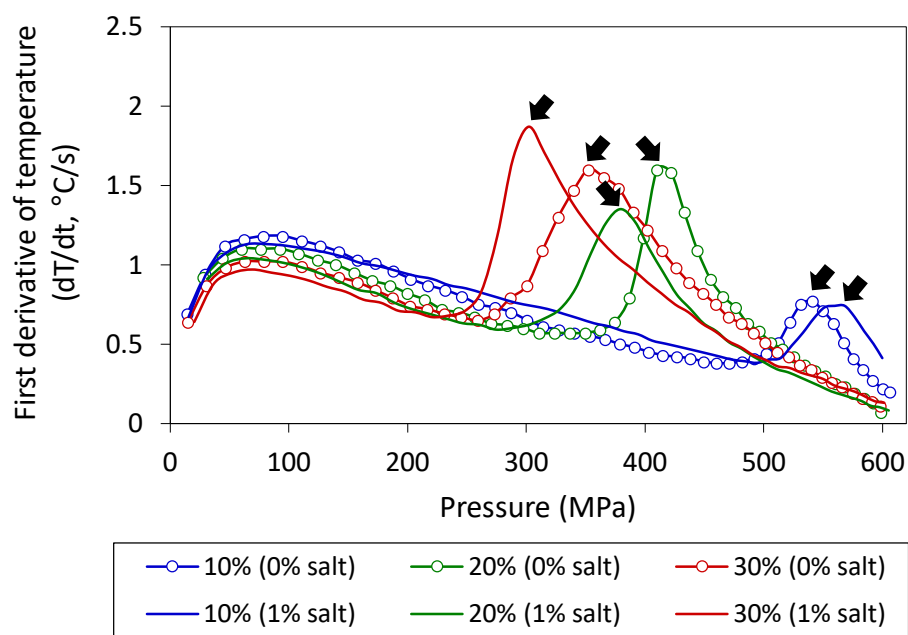

Supplement: Supplementary file 1 [file molecules-24-02853-s001.pdf]
